# Process Evaluation of a Time-Restricted Eating Intervention for Weight Management in South African Women Living with Overweight/Obesity and HIV on Dolutegravir-Based Antiretroviral Therapy: A Qualitative, RE-AIM-Informed Analysis

**DOI:** 10.3390/nu18030474

**Published:** 2026-02-01

**Authors:** Fatima Hoosen, Julia H. Goedecke, Joel A. Dave, Jonas S. Quist, Kristine Færch, Louise G. Grunnet, Amy E. Mendham

**Affiliations:** 1 Health Through Physical Activity, Lifestyle and Sport Research Centre (HPALS), Division of Physiological Sciences, Department of Human Biology, Faculty of Health Sciences, University of Cape Town, Cape Town 7700, South Africa; julia.goedecke@mrc.ac.za (J.H.G.); amy.mendham@sa.gov.au (A.E.M.); 2 Biomedical Research and Innovation Platform, South African Medical Research Council, Cape Town 7505, South Africa; 3 Division of Endocrinology, Department of Medicine, University of Cape Town, Cape Town 7925, South Africa; joeldave@endocrine.co.za; 4 Copenhagen University Hospital, Steno Diabetes Center Copenhagen, 2730 Herlev, Denmark; jonas.salling.quist@regionh.dk (J.S.Q.); louise.groth.grunnet.02@regionh.dk (L.G.G.); 5 Department of Biomedical Sciences, University of Copenhagen, 2200 Copenhagen, Denmark; 6 School of Psychology, University of Leeds, Leeds LS2 9JT, UK; 7 Department of Health Science and Technology, Faculty of Medicine, Aalborg University, 9260 Aalborg East, Denmark; 8 Riverland Academy of Clinical Excellence, Riverland Mallee Coorong Local Health Network, Berri 5343, Australia

**Keywords:** time-restricted eating (TRE), weight management, cardiometabolic health, obesity, low-resource setting

## Abstract

**Background:** South Africa faces the world’s highest HIV burden, disproportionately affecting women, alongside rising Type 2 Diabetes (T2D). Weight gain associated with preferred dolutegravir (DTG)-based antiretroviral therapy may worsen obesity and T2D risk. This process evaluation explored the implementation of a 12-month time-restricted eating (TRE) intervention for weight management in women with HIV and overweight/obesity in Khayelitsha, Cape Town. **Methods:** Using the RE-AIM framework, the study investigated the implementation journey. Data were collected from three groups: RCT participants, healthcare workers (*n* = 21), and fieldworkers (*n* = 3). Methods included structured informal interviews with TRE participants throughout the intervention and semi-structured in-depth interviews (IDIs) with a subset (*n* = 19) at 12 months. IDIs and focus group discussions were conducted with healthcare staff. **Results:** Implementation faced significant contextual challenges, including high food insecurity, economic constraints, and high crime levels. Cultural norms around food hospitality also posed barriers. Despite this, TRE was highly feasible and acceptable. Participants reported positive behavioural changes, establishing eating routines and consuming healthier foods. Perceived health benefits included improved appetite control, wellbeing, sleep, and weight management. Key facilitators were the intervention’s flexibility and, importantly, the non-judgmental, empathetic support from fieldworkers, which drove engagement and retention. Healthcare workers expressed willingness to integrate TRE into existing HIV counsellor-led services, and nearly all participants desired to continue TRE post-intervention. **Conclusions:** This process evaluation demonstrates that TRE is a contextually suitable and acceptable intervention from an implementation perspective. Its success in practice, however, depends on mitigating complex multi-level barriers through a flexible program design and high-quality, relationship-focused support integrated into existing healthcare infrastructure. **Trial registration:** PACTR202302484999720, 8 February 2023.

## 1. Introduction

South Africa faces intersecting obesity challenges: the world’s highest HIV burden, disproportionately affecting women [1], and rising type 2 diabetes (T2D) prevalence [2], with diabetes cited as the leading cause of death among women [3]. Within this context, concerns have emerged about weight gain associated with dolutegravir (DTG)-based antiretroviral therapy (ART), the preferred first-line HIV treatment, particularly for women [4]. This association is particularly concerning given its potential to exacerbate existing high obesity rates and, consequently, further increase T2D risk in this vulnerable population [5]. Traditional dietary interventions are challenging to implement in resource-limited settings due to widespread food insecurity [6]. Time-restricted eating (TRE), which limits the daily eating window rather than requiring specific foods or calorie restrictions, may therefore provide a feasible approach for weight management and improvement of cardiometabolic health in this setting.

The TESSA Study (Exploring the effects of Time-restricted eating on body weight and associated cardiometabolic outcomes in South African women) was a randomised controlled trial (RCT) designed to investigate the effects of a 12-month TRE intervention on changes in body weight and markers of cardiometabolic health in women aged 20–45 years, living with overweight or obesity and HIV on DTG-based ART, recruited from a community healthcare centre in Khayelitsha, Cape Town [7]. The intervention was co-designed with the target community after an extensive formative assessment phase [8], including a needs assessment and a pilot trial, to ensure cultural appropriateness and local contextual relevance.

Given the complex nature of the TRE intervention and its implementation in a historically disadvantaged, low-resource setting, a process evaluation is essential to address the “how” and “why” questions of implementation. This approach, as recommended by the UK Medical Research Council, assists in understanding why an intervention works or fails, how it can be optimised, and for refining underlying programme theory [9]. To guide this evaluation process, frameworks such as the RE-AIM Framework are utilized [10,11]. RE-AIM provides a structured way to measure results along these five dimensions, focusing on population-based measures to assess the effectiveness and transferability of the intervention from research into practice. The framework’s five dimensions include: Reach (the number, proportion, and representativeness of individuals willing to participate), Effectiveness (the impact on important outcomes), Adoption (the proportion and representativeness of settings and intervention agents willing to initiate the program), Implementation (the fidelity, consistency, time, costs, and adaptations of the intervention delivery) and Maintenance (the extent to which the intervention becomes institutionalized within settings and sustained by individuals over time) [10,11]. Complementing this is the Practical, Robust Implementation and Sustainability Model (PRISM) conceptual framework, designed to identify contextual factors that influence the successful implementation and sustainability of interventions [12]. PRISM systematically evaluates how the intervention interacts with its external environment, infrastructure, and recipients to influence key outcomes, explicitly guided by the RE-AIM framework.

The aim of this process evaluation was to explore the implementation journey of a 12-month TRE intervention in a low-resource setting, using the extended RE-AIM framework. We examined the perspectives of participants and key stakeholders to explore critical success factors, unforeseen challenges, and practical implications for implementation that are essential for successful project scalability in a comparable context. The quantitative effectiveness data (body weight and metabolic outcomes) will be presented in the forthcoming primary RCT results paper of the TESSA study, where these process evaluation findings will be integrated with quantitative outcomes data to explore mechanisms of impact as recommended by the MRC guidance. [9] This two-paper approach allows for comprehensive reporting of both the qualitative implementation insights (this paper) and the quantitative process-outcome linkages (forthcoming paper).

## 2. Materials and Methods

This paper has been structured according to the Consolidated criteria for Reporting Qualitative Research (COREQ) Checklist [13].

### 2.1. Study Design

This process evaluation aimed to understand the barriers and facilitators of a 12-month TRE RCT for sustainability in a low-resourced setting, using the extended RE-AIM framework, accounting for contextual factors. The TRE intervention involved restricting the daily eating window to 8–10 h, personalised to participants’ habitual eating patterns and supported by nutritional information sessions and alternating weekly phone calls and text messages. Participants attended monthly study visits throughout the 12-month intervention period. Major assessment visits (baseline, 3-, 6-, 9-, and 12-months) were conducted off-site from the Ubuntu ART clinic due to space constraints, while interim monthly visits were conducted at the clinic. The TESSA study design is described in detail elsewhere [7].

### 2.2. Participants

The process evaluation included three participant groups, namely the TESSA RCT participants, healthcare workers, and the fieldworkers of the RCT. This study was performed in collaboration with the Ubuntu ART Clinic at Site B Community Health Centre in Khayelitsha, Cape Town, from where all participants were recruited. This clinic serves a large population of approximately 12,500 patients on ART. Khayelitsha is a vast peri-urban informal settlement where the community are predominantly (99%) *isiXhosa*-speaking, Black South Africans [14].

*RCT participants:* Inclusion criteria included women, between 20–45 years of age, living with HIV and having initiated DTG-based ART within the previous 1–24 months, and a BMI ≥ 25 kg/m^2^. Recruitment took place in the waiting area of the Ubuntu ART Clinic, which medically stable patients typically visit every two months for medication refills. Throughout the 9-month recruitment period, a fieldworker stationed at the clinic described the study’s purpose and inclusion criteria in *isiXhosa* to patients in the waiting area. Methods of recruitment included approaching patients one-on-one or in groups, and “snowballing”, where eligible participants recruited family and friends. These announcements were made every two hours to capture new patients as they arrived. Interested individuals provided their contact details to arrange screening appointments at the clinic. Upon screening, eligible participants received further written information regarding the study.

*Healthcare workers:* Healthcare workers based within the Ubuntu ART Clinic at Khayelitsha were recruited, and included a medical doctor, a clinical nurse practitioner and HIV counsellors.

*Fieldworkers:* The TESSA study fieldworkers included a registered nurse and two research assistants.

### 2.3. Data Collection

*Socio-demographic data:* Data was collected as per the TESSA study protocol [7].

*RCT participant interviews:* As part of the TESSA study protocol, face-to-face, structured informal interviews were conducted with participants randomized to TRE (*n* = 47 at baseline) every 3 months (3 months, *n* = 37; 6 months, *n* = 34; 9 months, *n* = 31; 12 months, *n* = 35). Questions were developed by AEM and FH, and explored changes implemented, reflections on TRE, family support, recommendations and willingness to continue with the study (Supplementary Material S1). A fieldworker conducted the interviews in a private room at the study site and entered the participant responses into REDCap (Research Electronic Data Capture, Vanderbilt University, Nashville, TN, USA [15]. In addition to these structured interviews, participants were further invited to participate in semi-structured in-depth interviews (IDIs) at the end of the 12-month TRE intervention, allowing for more detailed feedback regarding their experiences. This interview guide was developed by FH and JHG (Supplementary Material S1), pilot tested with existing TESSA participants (*n* = 2), and as no major revisions were required, the interviews were included in the final sample (*n* = 19). IDIs were either conducted face-to-face in a private area at the study site (*n* = 8) or telephonically (*n* = 11), and lasted 20 to 30 min. Although attempts were made to contact those lost-to-follow-up, we were unable to recruit any for re-engagement. Data collection continued until saturation was reached, defined as no new themes emerging from the data. Both informal interviews and IDIs were conducted in *isiXhosa* by trained fieldworkers, with FH providing oversight, between July 2023 and December 2024.

*Healthcare worker interviews:* Face-to-face interviews were conducted with healthcare workers at the Ubuntu ART Clinic prior to commencement of the RCT (October 2022) by AEM in English. AEM developed the questions, which explored perceptions regarding the need for a weight management programme and its adoption within the existing clinic setting (Supplementary Material S1). IDIs were conducted with the doctor and the clinical nurse practitioner, lasting approximately 30 min each and two focus group discussions were conducted with the HIV counsellors (*n* = 8; *n* = 13), lasting approximately 60 min each.

*Fieldworker interviews:* FH conducted face-to-face IDIs with the TESSA study fieldworkers. These were administered in English at the study site at the conclusion of the RCT, December 2024, and lasted between 45 to 60 min. FH and JHG developed the questions which investigated perceptions regarding their experiences in the implementation process of the intervention (Supplementary Material S1).

During all interviews, observational notes were taken to document non-verbal cues and salient points, and a summary was provided to participants following each interview for their review to ensure accuracy.

*Meeting notes:* Weekly meetings were conducted with the local South African research team which included fieldworkers and researchers, while monthly meetings were conducted with the larger team, including international collaborators. Meeting minutes were written and used to monitor and track any changes adopted during implementation of the project.

### 2.4. Qualitative Data Analysis

Participant responses from the structured interviews were extracted from the REDCap database. All other interviews were audio-recorded, translated into English where needed and transcribed. All data were analysed using NVivo qualitative data analysis software (Version 14, QSR International, Melbourne, Australia). Interview transcripts were analyzed using thematic analysis, following the six-phase, iterative approach outlined by Braun and Clarke, encompassing data familiarization, coding, thematic framework development, review, theme definition with interpretation writing up the report [16]. This method was selected for its flexibility in identifying patterns of meaning across the dataset while allowing both inductive and deductive analysis. FH conducted the initial inductive theme identification, which was subsequently mapped deductively against the RE-AIM domains.

Inter-rater reliability was maintained through AEMs quality assurance review of all code designations, quotations, and thematic mappings. Discrepancies were resolved through collaborative discussion of coded transcripts between researchers until consensus was achieved. Categorical data frequencies were calculated, while continuous descriptive data are presented as means and standard deviations or medians and 25–75th percentiles, depending on the data distribution.

AEM and JHG are experienced researchers, and FH is a postdoctoral fellow; all three are female and have previous qualitative research experience. The fieldworkers who conducted the participant interviews were all trained and have extensive experience. The informal interviews were conducted by a male, while a female conducted the remaining focus group discussions and IDIs.

## 3. Results

### 3.1. Characteristics of RCT Participants

Table 1 presents the sociodemographic characteristics of the TESSA cohort. The mean age (min-max range) was 37 (21–45) years and ART duration (min-max range) was 13 (1–23) months. Nearly all participants were of isiXhosa ancestry (99%). Most lived in informal housing (56%) and were unemployed (67%), with only 11% of women having completed high school or tertiary education. Severe food insecurity was prevalent among most participants (59%), with higher rates observed in the TRE group compared to the control group (70% vs. 48%).

### 3.2. Process Evaluation According to RE-AIM

**Figure 1:**
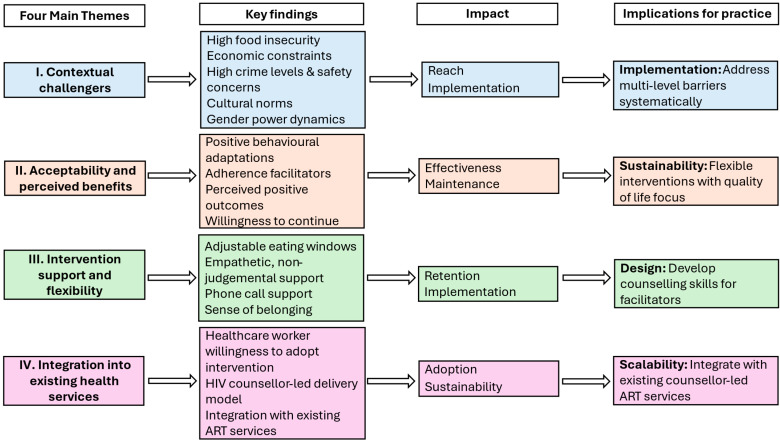
The Four Main Themes Emerging Across the RE-AIM Dimensions: Synthesis of the Time-Restricted Eating Intervention Process Evaluation. ART: Antiretroviral therapy.

The results describe the process evaluation of the 12-month TESSA RCT across the RE-AIM dimensions of reach, effectiveness, adoption, implementation and maintenance. Figure 1 presents the four main themes that emerged across these dimensions, namely contextual challenges, high acceptability and perceived benefits, intervention support and flexibility, and integration into existing health services.

#### 3.2.1. Reach

We recruited 404 women; however, 293 were excluded at pre-screening and 18 at screening, resulting in 93 eligible participants—falling short of our target of 152.

Recruitment involved fieldworkers establishing collegial relations with healthcare workers at the Ubuntu ART clinic, then approaching patients individually or in groups in the waiting area. “Snowballing”, where participants referred family and friends, was also employed. These methods generated considerable interest, as evidenced by the high initial numbers. However, strict eligibility criteria, notably age limits and pre-existing conditions like hypertension, resulted in substantial exclusions.

Among the 93 recruited, 23% (n = 22) were lost-to-follow-up at 12 months. Fieldworkers observed that self-referred participants showed higher attrition rates, which they attributed to financial motivation over genuine study interest: *“Yeah. But those ones who approached us on themselves were maybe looking for a study, not for… I’m saying maybe they were looking for benefits from the study, not the study itself… The benefit like a stipend that we get from the study.”* (FW 001). Reported reasons for lost-to-follow-up included participants becoming uncontactable, depression, and pregnancy.

#### 3.2.2. Effectiveness

*Behavioural adaptations:* The most common behaviour change reported by participants, throughout the intervention, was dietary changes which included establishing an eating routine and adhering to the eating window, decreased fat intake, increased fruit and vegetable intake, increased water intake, mindful eating, and portion control: *“When I started on TRE I was eating whatever I could find and whenever I want to eat. But since I have adopted the TRE I try to eat within my time (restricted) eating window.”* (PID 22, 12 months); and *“I started eating healthy. Try to avoid fat things I reduced my salt intake and eating on my window scheduled. Yes!.”* (PID 391, 12-month). Many participants reported developing greater self-discipline, particularly in managing hunger cues and adhering to their eating window: *“I would feel hungry and want to eat there and then. But now there is a huge difference because I can control myself.”* (PID 179, 12 month). Additionally, fieldworkers perceived that participants learnt to recognise cues for hunger vs. eating when bored. While a few participants reported increases in physical activity, fieldworkers highlighted significant contextual barriers to its widespread adoption, including high crime levels and a social stigma that frames exercise as a difficult task.

*Perceived outcomes:* Participants perceived multiple positive outcomes in response to TRE adoption which included having an improved regulation of appetite, a decreased feeling of hunger, an improved feeling of wellbeing, and most perceived that their weight had either decreased or been maintained or that they had a smaller body size, including a flatter tummy. Some quotes illustrating these perceptions included the following: *“My appetite has one hundred percent improved and I can now control it.”* (PID 318, 12 month) and *“My body has becoming lighter and lighter ever since I joined (the) TRE program. I don’t get exhausted easily like before. My sleep has also improved. I feel my health is controlled.”* (PID 227, 6 month).

Although some participants reported their weight had increased, their overall perception about the intervention remained positive: *“I have accepted myself thanks to you guys and this study. I feel normal like any other person. I share more with my family and we are happy. I am happy with my appetite. I have gained a little weight though. Otherwise I am happy.”* (PID 164, 12 month).

*Facilitators to adherence*: Many participants reported a heightened awareness of their health because of the intervention, which was closely linked to treatment adherence: *“Also, your program can help to encourage people to stick to their treatment. When someone from the club can join the study, she can learn many things about her health and how to maintain healthy lifestyle and adhere to the treatment that is given at the clinic”* (PID 043, 12 month). One participant suggested that adopting TRE improved her ART adherence, while another reported that reflecting on the health implications of overeating helped her to resist food cravings. It was considered helpful that TRE only restricted eating times and not specific foods. Perceived ease of adherence was a key facilitator, with both participants and fieldworkers reporting TRE became easy after initial habituation: *“In the beginning it was challenging to get used to eating with the set time window but as time went by, I got used to the program.”* (PID 375, 12 month).

*Social and family-related factors:* Family support emerged as a key factor influencing TRE adherence. Family members were mostly supportive of participants following TRE, with some adapting their eating times to align with participants’ eating windows or preparing food for later consumption. Some family members adopted TRE themselves or expressed an interest: *“My boyfriend likes food, but he is trying to adapt to my eating times.”* (PID 318, 12 month). Participants who shared their TRE journey with family members reported improved relationships: *“My family relationship has greatly improved. We are now open to each other. We talk about the healthy foods that we are supposed to eat. We talk about avoiding sugary foods and salt. They have been supportive of me on TRE and they know about my status.”* (PID 349, 12 month).

However, unsupportive family members posed barriers and managing misaligned eating times was challenging: *“If members of the household would wake up in the early morning to have breakfast before my eating time window starts, I wouldn’t join in, I would wait for my eating time window to start for me to eat…Yes, it would be difficult.”* (PID 010, 12 month). Some participants concealed their TRE participation from family. Fieldworkers attributed this to fears that TRE would be associated with HIV status or cultural stigma associated with weight loss: *“When we introduced the study, we asked them if they were living with HIV and if they were on the pill (ART)… That one, we explained it, but they could still attach it (TRE) to HIV…Another thing is some of their family members have this stigma about losing weight. This is attached maybe culturally, that thinks that if you lose weight, you cease to be that African person.”* (FW 001).

Fieldworkers also identified abusive relationships and partners control over participant’s involvement in the study: *“Some who were married were really restricted by their partners. Their partners, uhm I don’t want to mention this, but I found out that, or maybe I can impute this, because it was not told to me, but from the interaction I could pick it up, that some participants were living in abusive relationships…And it was a challenge for the member to stick to this program on the time-restricted eating for that one.”* (FW 001). This controlling behaviour sometimes extended to specific study procedures; for example, one male partner objected to a male fieldworker contacting his female partner.

Other social barriers included family visits outside of Cape Town, as participants navigated undisclosed HIV status or struggled to adjust eating times with family schedules. Cultural expectations around food hospitality presented particular challenges, with the fieldworker explaining that food is central to social gatherings and having large portions signifies hospitality. Not eating at family events was considered both taboo and impolite: *“We eat in our culture, food is, in any culture for that matter, food is what brings people together… there is always food. It is such a taboo not to eat. You know. When you say no thank you; you are not gonna eat, that is considered not so polite.”* (FW 003). Fieldworkers suggested practical solutions like bringing containers to pack food for later consumption while other strategies employed by participants included drinking water or black coffee during non-eating periods and focusing on conversation.

*Environmental and economic factors:* Fieldworkers identified multiple environmental challenges specific to the Khayelitsha context, including high levels of food insecurity. This made refusing available food particularly difficult for participants. Meat offerings were perceived as especially enticing and difficult to decline and represented a missed opportunity to access food not often enjoyed. Participants reported increased cravings when food was scarce at home. Economic barriers compounded these challenges, as local fruit and vegetable prices are prohibitively high while transport costs to access better-quality food elsewhere were unaffordable. Employment status emerged as another important factor, with participants reporting frequent changes throughout the study period. Being employed was associated with an improved dietary intake while being unemployed or any other loss of income (such as a bursary loss) resulted in a limited and less healthy diet: *“Because I am working now, there is a lot that has changed in our diet at home. We can now afford to buy some vegetables and fruit which we couldn’t afford when I was unemployed.”* (PID 349, 6 months). Work schedules presented both facilitators and barriers to TRE. Employment could structure the day and aid adherence, as one participant noted: *“It has been even better now that I am working because I would delay eating or even don’t feel hungry until my eating time window.”* (PID 365, 12 months). Conversely, unstable work conditions, such as relocating for new employment opportunities, disrupted routines and made adherence difficult, while working night shifts posed a significant challenge to complying with follow-up visits.

#### 3.2.3. Adoption

The clinical nurse practitioner reported that the capacity to accommodate the large, increasing number of patients presenting with both HIV and non-communicable diseases is currently severely limited at the clinic. There are three integrated clinic clubs to accommodate patients with HIV and hypertension, where only 90 patients can be supported: *“We cannot accommodate everybody. Because at… (the clinic) ….I think we got more than 18,000 patients.”* (HCW 002). The healthcare workers felt that a TRE intervention would be well-supported by their colleagues, suggesting it be integrated into the existing clinic setting, led by the HIV counsellors during their scheduled bi-monthly appointments with medically stable patients collecting their prescriptions. The counsellors perceived it would be easy for them to implement as they have previously provided similar support to patients and they have existing good relations with the patients who trust them: *“I think when we do the education, then we can introduce this fasting thing and then motivate them.”* (HCW 003)). The nurse recommended that nursing staff train the counsellors, using a training package (as was previously done) and reading material for the participants. The counsellors perceived that more space would be needed in the clinic and they would need mobile phones to remind patients about their eating windows. Participants also recommended that the TRE intervention be integrated with the usual HIV care in the clinic setting.

#### 3.2.4. Implementation

*Study site:* Due to space constraints at the Ubuntu ART clinic, baseline visits were conducted at a research laboratory at the University of Cape Town. However, this presented a significant barrier as participants found the commute from their homes in Khayelitsha to this facility to be long and difficult. Consequently, the study site moved to a church within the Khayelitsha area. However, lack of security at this site resulted in the team being robbed at gunpoint, with research equipment and personal belongings stolen. For the safety of participants and the research team, the study site was forced to move for a third time to a tertiary hospital in Cape Town. Although still far from Khayelitsha, it was easier for participants to travel to from their homes than the research laboratory.

During the shorter monthly visits at the clinic, fieldworkers reported running from room to room with participants and equipment looking for a free space. This was especially a challenge during the very busy morning periods: *“It is the space, also in the facility, but there are times that I have run around…with the client because…We do not have proper space, where uh…sometimes I move with the scale running and looking for the space.”* (FW 002).

*Follow-up visits:* Fieldworkers identified many barriers with follow-up visit compliance. Mondays, grant days (when social assistance payments are distributed), rainy days and pay days were especially difficult. Participants securing employment presented another challenge to attending follow-up visits. It was also difficult to contact participants if they did not have their own personal phones or had lost their phones. In these cases, the team attempted to maintain contact by calling neighbours or partners.

Environmental factors also impacted follow-up visits. Many participants (including fieldworkers) were affected by the winter floods where personal possessions and even entire homes were lost. Additionally, taxi protests made it unsafe for participants and fieldworkers to travel to the study site. Although the team felt it was insensitive to request participants to attend follow-up visits during these difficult times, they maintained contact with them—an effort that was appreciated: *“The fact that we didn’t pressure them, we stick to calls, how are you doing, and they appreciated them, (saying) Oh, my word you are the first person to call to ask how I am doing!”* (FW 003). As many participants lost their phones, the only way they could maintain contact with the team was by physically attending the clinic, where they knew a team member was permanently stationed. Lack of safety in the area made participants too afraid to walk to appointments, with one participant experiencing an attempted rape and another being robbed, en route to the clinic. Theft of phones and self-monitoring calendars created additional barriers.

*Data capturing:* All RCT data were captured electronically into REDCap, enabling a virtual calendar where all intervention appointments and events were tracked, which the fieldworkers found to be very effective. However, an electronic system also had challenges. The clinic had poor internet access, and this coupled with fieldworkers being too afraid to travel with their electronic devices due to safety concerns, meant that entering data in real-time was a challenge. Consequently, data was recorded on paper and then later entered into REDCap either by the original fieldworker or another fieldworker on their behalf. Fieldworkers also reported that there were times when equipment was not functional, resulting in the use of equipment other than that assigned to the intervention, admitting that the calibration of this equipment was not known.

*Self-reported data:* Fieldworkers perceived that compliance with completion of the daily, self-monitoring calendar became difficult over time, with participants becoming bored, and questioned the accuracy of its completion as calendars with perfect eating windows with no variability were presented: *“Our first participants they find it uh, interesting, and they find it so easy, and they were doing it correct because you see on the calendars they were writing 5 past eleven when they start at 11:00 and then in the end of the intervention I think they get bored.”* (FW 002). The fieldworkers also raised concerns about the accuracy of other self-reported data, including fasting status prior to blood draws, and the potential influence of social desirability bias on responses.

*Intervention delivery:* The fieldworkers found that coordinating the eating window with the timing of ART worked well, even improving ART adherence. They also found that a flexible approach was beneficial, such as adapting eating windows to accommodate participants’ changing circumstances like employment changes. Both participants and fieldworkers perceived that the phone call was the best method of support provided by the intervention. It allowed for check-ins to occur and subsequent adaptations to be made as needed without delays, motivated the participants and served as a reminder of appointments. Some participants and fieldworkers also suggested that WhatsApp groups could be a valuable future tool for support. A fieldworker also mentioned that having a member of the team permanently based at the clinic was helpful to maintain the link between the team and participants.

Other supportive strategies employed by the fieldworkers included being kind, providing continuous encouragement, listening to their many personal problems without passing judgement and providing counsel or referring them to a counsellor if needed. They endeavoured to make participants have a sense of belonging. Some participant quotes reflecting the effectiveness of these strategies and how this translated into practice include: *“It is the phone calls and the support I get from the program team, it helped me to stay in the program and not leave…It is because the team members are kind, they love the people, they speak with respect to us, and they encourage you to stay with the program. I don’t feel alright now that the program is complete because I will miss the team.”* (PID 182, 12 month) and *“I got much support. You have been a very friendly team. You allowed us the space to talk about many other things, let alone the dietary advice. Yes.”* (PID 220, 12 month). Despite the many information and education sessions, it emerged that some participants had difficulty understanding the purpose of the intervention or the concept of observing an eating window. In addition, participants had expectations beyond the scope of the study, where some expected to be taken to a gym and others expected to receive food parcels. The fieldworkers managed these situations by breaking down information, providing clear instructions, having patience, showing understanding and repeating information. They also felt that speaking in the same language as the participants was helpful.

#### 3.2.5. Maintenance

Although no follow-up visits were conducted after the 12-month visit, all participants were asked about their willingness to continue with TRE, with most responding positively. Reasons provided for this included that participants felt a sense of control over their health, their family were supportive of them adopting TRE, they recognised the benefits associated with TRE, they had become accustomed to it and it was considered something that worked for them. Participants requested that a support group be started to ensure that they could continue with TRE after the intervention ended. Some quotes reflecting participants’ willingness to continue include: *“Even now after this program, I will continue to eat in the eating time window that you gave me, because I would want to be fat again. I am comfortable with my weight the way it is now.”* (PID 365, 12 month) and *“I will continue eating in my eating time window, I will continue eating healthy and stay healthy…I want to keep on maintaining a healthy lifestyle. If I stop doing what I am doing now, it will be to my disadvantage. The way I am now is the right one for me; I eat healthy and on time, I exercise. I will continue that way.”* (PID 386, 12 month).

## 4. Discussion

This process evaluation, guided by the RE-AIM framework extended by PRISM, provides important insights into the implementation of a 12-month TRE intervention within a low-resource community in Khayelitsha, Cape Town. By exploring the perspectives of participants, healthcare workers, and the research team, we identified how the intervention’s reach, perceived adoption, implementation, perceived effectiveness, and potential for maintenance and sustainability were shaped by four overarching themes: (1) contextual challenges; (2) high acceptability and perceived health benefits; (3) the support provided and flexibility of the intervention design; and (4) the feasibility of integration into existing health services. A summary of findings mapped directly to the RE-AIM dimensions is provided in Figure 1. Despite significant contextual barriers, the TRE intervention was feasible and acceptable, with its success dependent on a flexible intervention design, empathetic support from the research team, and leveraging existing counsellor-led service delivery.

The Khayelitsha setting profoundly shaped all aspects of the TRE intervention, presenting a complex interaction of economic, structural and social barriers, demonstrating how the broader social determinants of health in low socioeconomic communities severely restrict the ability to engage in healthy behaviours and access services [17]. Economically, high levels of food insecurity made adhering to TRE difficult when participants had to refuse available food during their fasting periods. highlighting the tension between adherence to the intervention and survival needs. Additional economic barriers further exacerbated these issues, with prohibitively high local food prices and unaffordable transport costs to access better-quality food elsewhere. This led to opting for more affordable, often unhealthy foods rather than healthier options, a common challenge in low-income communities [18,19]. Another implication of financial constraints is that the stipend, while necessary to facilitate participation, may have incentivised enrolment for some more than a genuine interest in weight management. This lower intrinsic motivation is a known risk factor for higher attrition and lower engagement and adherence to interventions [20].

Safety and environmental factors presented significant structural barriers. High crime levels limited opportunities for physical activity, while the fieldworkers themselves faced threats, including being robbed at gunpoint at an unsecured study site, forcing multiple site changes. Furthermore, environmental events such as winter floods and taxi protests severely disrupted follow-up visits, making travel unsafe for both participants and fieldworkers. Participants also reported an attempted rape and robbery on their way to the clinic, leading to fear and further non-attendance. These realities underscore the severe limitations that such an environment places on health interventions and daily life, reflecting broader inequalities in physical environment, housing, and access to resources [17].

Socially, cultural factors emerged as critical influencers of TRE adoption. Family support was found to be both a facilitator when present, while its absence acted as a barrier, a common finding in TRE interventions [21,22,23,24]. The intervention, at times, positively transformed household dynamics, with family members not only providing support but actively adapting their own schedules to align with the participant’s eating window. In several cases, the shared focus on healthy eating fostered by TRE improved family communication and relationships, creating a supportive home environment. This demonstrates that the intervention, when supported, could integrate successfully into the fabric of daily family life. Differing eating schedules and cultural norms regarding food hospitality presented barriers, underscoring the need for tailored strategies to involve and educate households [22]. The influence of gender power dynamics was particularly salient, extending beyond simple disapproval. Some women found themselves in restrictive situations that undermined their self-determination, aligning with findings of women in similar low-income contexts [20]. These factors illustrate that dietary behaviour changes are not individual acts but deeply social ones that interact with—and can be derailed by—powerful cultural norms and power dynamics [25].

Equipment and data management issues such as non-functional equipment, real-time data entry challengers and self-reported data bias [26], impacted smooth implementation of the RCT. These challenges should therefore be considered when interpretating certain quantitative outcomes, and highlight the need for robust, yet context-appropriate, monitoring strategies. This detailed understanding of the local socio-cultural landscape, including its potential as a facilitator, is a central strength of this study and provides a critical blueprint for future implementation.

The perceived positive outcomes reported by participants were overwhelming and a major driver of engagement. The value they placed on these perceived benefits, such as increased feeling of wellbeing and improved appetite control, far exceeded the focus on weight management alone. Additionally, TRE principles do not restrict specific foods, enhancing its appeal and feasibility in a context of widespread food insecurity. Despite participants acknowledging this benefit, many chose to improve their dietary intake, in accordance with the provided education sessions, which was a direct result from the co-design approach employed during the formative phase of the TESSA study. This voluntary behavioural adaptation, which included implementing portion control and consuming more fruits and vegetables, resulted from the heightened health awareness gained through the intervention, which participants used to actively manage cravings and develop self-discipline. This knowledge-to-behaviour translation represents an essential step in behaviour change consistent with Health Belief Model principles [27]. This suggests that the success of a health intervention in such challenging contexts may depend less on achieving specific clinical targets and more on delivering holistic health benefits that translate into tangible, daily improvements in quality of life that participants immediately recognise and value [28]. This is the most plausible explanation for the overwhelming desire to continue TRE post-trial, making it a powerful indicator of acceptability and a cornerstone for potential sustainability [29].

Despite the challenging context, the TRE intervention’s inherent design features were key facilitators. The intervention’s adaptable design—it’s personalized and adjustable eating windows—functioned as a direct counter to the rigid barriers of the external environment. For a participant dealing with an ever-changing employment status, having a flexible eating window can be the difference between adherence and drop-out. This ability for quick, micro-level adjustments allowed for a pragmatic, person-centred approach where context is a core design feature of the intervention [15].

This process evaluation highlighted the critical role of reliable support systems and relational dynamics in promoting engagement and adherence. The fieldworkers were consistently praised by participants who exceeded their prescribed duties to become confidants and advisors, providing the non-judgmental support necessary to navigate a challenging environment. Their empathetic approach fostered a strong sense of belonging, which was important for retention. These findings are supported by literature on the importance of relational continuity and trust and its positive effect on health outcomes [30], even in primary care settings [31]. A key finding was the potential for a synergistic relationship between TRE and ART adherence. Fieldworkers reported that coordinating the TRE eating window with the timing of DTG-based ART intake was not only feasible but often served as a reinforcing cue, which participants and fieldworkers perceived as potentially improving adherence to both regimens. This alignment of a new health behaviour (TRE) with an established ART regime is a significant advantage in this clinical population and should be a core component of future implementation. Phone calls, a well-known behaviour change technique employed to improve dietary intake [32], were perceived as the most effective method of support, allowing for timely check-ins, adaptations, and appointment reminders.

Importantly, healthcare workers expressed willingness to support and integrate TRE into the clinic setting, specifically the counsellor-led service. This aligns with implementation science that interventions are more likely to be sustained when adopted by trusted front-line staff like counsellors [33]. This strategy of integration also conforms to the PRISM framework, which emphasises implementing interventions within existing infrastructure and care teams to improve compatibility, avoid creating new service demands, and ensure sustainability [12]. Furthermore, the significant challenge of dedicated physical space, which hindered data collection during the research phase, is likely to be mitigated upon full integration into routine care. This represents a low-cost, high-compatibility addition essential for scaling across other overburdened, low-resource health systems. Healthcare workers suggested that training packages and reading materials could facilitate this integration, with nursing staff potentially training counsellors. This demonstrates sustainability through perpetual training and capacity building without requiring external support. The ultimate indicator of national success would be the formal inclusion of TRE into national policy guidelines for managing weight among women living with HIV.

We propose the following recommendations for future practice and research:

1. *For program designers:* TRE interventions should be designed with built-in flexibility and should incorporate a strong component of training on relational and counselling skills for facilitators;
2. *For policy makers and implementers:* Scaleup is feasible and requires a multi-level approach. TRE education should be provided at an individual level; family engagement strategies should be employed at an interpersonal level; space and capacity constraints should be evaluated and addressed at a clinic level; and the impact of food security and safety must be acknowledged and mitigated;
3. *For researchers:* Future studies should investigate the specific role of facilitator-participant relationships on outcomes and test the effectiveness of TRE when fully led by integrated clinic counsellors rather than research teams, with a focus on longer-term studies.

A significant strength of this process evaluation is the rigorous application of the RE-AIM framework, extended by PRISM, which provided a robust and comprehensive lens for understanding the intervention’s complexities within its specific context. The triangulation of data from multiple stakeholders—RCT participants, various healthcare workers, and the research team—offered a rich, multifaceted understanding of implementation from diverse perspectives. However, limitations must be acknowledged. This includes the potential for social desirability bias in interviews, though several strategies were employed to minimize this risk: the interviewer had established rapport with participants prior to the interviews, and explicit assurances of confidentiality and anonymity were provided. Furthermore, we were unable to interview participants lost-to-follow-up, which may mean we missed the most negative experiences and barriers. Although the TESSA study was conducted at a single healthcare facility, the implementation lessons are highly transferable and directly relevant to other low-resource, overburdened health systems [34]. Following MRC guidance [9], a complete process evaluation includes both qualitative exploration of implementation (reported here) and quantitative analysis linking process data with outcomes to explore mechanisms of impact. The latter analysis—examining how process variables such as adherence, fidelity, and contextual factors relate to weight and metabolic outcomes—will be reported in the primary RCT results paper. This two-paper approach ensures comprehensive coverage of both components without compromising the depth of either analysis.

## 5. Conclusions

This process evaluation of a TRE intervention for women living with HIV in South Africa reveals that while TRE is a contextually suitable weight management strategy, its success in practice is significantly mediated by socio-ecological factors, relational dynamics, and the quality of implementation support. Despite these challenges, the intervention demonstrated high acceptability and perceived health benefits, leading to a strong desire for maintenance.

## Figures and Tables

**Table 1:** Sociodemographic characteristics of the TESSA cohort.

	All Participants (*n* = 93)	TRE (*n* = 47)	Control (*n* = 46)
Age, years	38 (9)	39 (7)	36 (8)
TLD treatment duration, months	13 ± 4	13 ± 4	13 ± 5
**Ethnicity, *n* (%)**			
Xhosa	91 (99%)	45 (98%)	46 (100%)
Sotho	1 (1%)	1 (2%)	0
**Education, *n* (%)**			
Grade 1–7 (Primary School)	3 (3%)	2 (4%)	1 (2%)
Grade 8–12 (High School)	79 (86%)	38 (83%)	41 (89%)
Completed high school or tertiary education	10 (11%)	6 (13%)	4 (9%)
**Employment status, *n* (%)**			
Employed	30 (33%)	13 (28%)	17 (37%)
Unemployed	62 (67%)	33 (72%)	29 (63%)
**Socioeconomic status**			
Formal/Brick house, *n* (%)	40 (44%)	15 (33%)	25 (54%)
Informal/Shack, *n* (%)	52 (56%)	31 (67%)	21 (46%)
Housing density (number of people/room)	1 (1, 2)	1 (1, 2)	1 (1, 2)
Asset Index (*n*/12)	6 (5, 7)	6 (5, 7)	6 (5, 8)
Food security			
Food secure	7 (8%)	4 (9%)	3 (7%)
Mild food insecurity	9 (10%)	3 (6%)	6 (13%)
Moderate food insecurity	22 (24%)	7 (15%)	15 (33%)
Severe food insecurity	55 (59%)	33 (70%)	22 (48%)

Normally distributed data is presented as mean (SD) and skewed data is presented as median 25th, 75th percentile. Categorical data are reported as n (%). Abbreviations: Tenofovir, Lamivudine, and Dolutegravir, TLD.

## Data Availability

The data presented in this study are available upon reasonable request to the corresponding author, pending ethical approval from the Faculty of Health Sciences Human Research Ethics Committee, University of Cape Town.

## Supplementary Materials

The following supporting information can be downloaded at: https://www.mdpi.com/article/10.3390/nu18030474/s1, Supplementary Material S1: Interview guides.

## Abbreviations

The following abbreviations are used in this manuscript:

T2D	Type 2 Diabetes
DTG	Dolutegravir
TRE	Time restricted eating
ART	Anti-retroviral therapy
TESSA	Exploring the effects of Time-restricted eating on body weight and associated cardiometabolic outcomes in South African women (The TESSA Study)
RCT	Randomized controlled trial
RE-AIM	Reach, Effectiveness, Adoption, Implementation, Maintenance Framework
PRISM	Practical, Robust Implementation and Sustainability Model
COREQ	Consolidated criteria for Reporting Qualitative Research Checklist
REDCap	Research Electronic Data Capture, Vanderbilt University, Nashville, TN, USA
IDI	In-depth interview
TLD	Tenofovir, Lamivudine, Dolutegravir
PID	Participant identity
HCW	Healthcare worker
FW	Fieldworker
