# Supplementary material for: Process Evaluation of a Time-Restricted Eating Intervention for Weight Management in South African Women Living with Overweight/Obesity and HIV on Dolutegravir-Based Antiretroviral Therapy: A Qualitative, RE-AIM-Informed Analysis
Source: Nutrients. 2026 Feb 1;18(3):474. doi: 10.3390/nu18030474 (PMC12899678; doi:10.3390/nu18030474)
Supplement: Supplementary file 1 [file nutrients-18-00474-s001.zip › nutrients-4000099-supplementary.pdf]

Interview guide at 3, 6, 9, and 12 months for TESSA participants

1. Have you changed anything in your life (exercise, diet, medication, work?)
2. What have you changed?
3. Can you reflect on the last 3 months and tell us if you have noticed any changes in your body, health, appetite?
4. Is your family/partner also following TRE? Please provide details.
5. Are there any changes or recommendations that you would like to suggest for the project?
6. Are you willing to continue with the project? Can you please explain why?

12-month in-depth interview Guide for TESSA participants

Opening Questions

1. "Can you tell me about your overall experience with the eating program?"
   - How long did you participate?
   - What made you decide to join initially?

Understanding Dropout

2. "Could you tell me about what led you to stop participating in the program?"
   - What was happening in your life at that time?
   - Was there a specific event or moment that made you decide to stop?

Daily Challenges & Experiences

3. "What parts of the program were difficult for you?" Probe for:
   - Managing eating times
   - Taking medications
   - Family/social situations
   - Work schedule
   - Transportation
   - Health issues
4. "How did the program affect your daily routine?" Probe for:
   - Morning schedule
   - Work/school timing
   - Family meals
   - Social activities

Positive Aspects

5. "What parts of the program worked well for you?" Probe for:
   - Easy aspects
   - Helpful resources
   - Support received
   - Any benefits noticed

Support Systems

6. "Tell me about the support you had while in the program?"
    - How did your family/friends react?
    - How was your experience with the program staff?
    - What additional support would have helped?

Suggestions

8. "What could we change to make the program work better for you?"
    - What would have helped you stay in the program?
    - What other support would you have liked?

Closing

9. "Is there anything else you'd like to share about your experience?"

Thank you for your feedback! Your answers will help us improve the program for others.

Interview guide for TESSA Fieldworkers

1. Study Participation and Context

    - Can you describe your role in the time-restricted eating study?
    - What was your understanding of the study's primary objectives/goals?
    - How did you initially approach participant recruitment in Khayelitsha?

2. Participant Engagement and Adherence

    - What were the most significant challenges you observed in participants maintaining the time-restricted eating protocol?
    - How did participants initially respond to the intervention?
    - Can you share examples of how participants adapted to the eating time restrictions?
    - What methods did you use to support participant adherence?

3. Cultural and Contextual Considerations

    - How did local cultural practices and daily routines impact participants' ability to follow the time-restricted eating protocol?
    - What unique challenges did you encounter specific to the Khayelitsha community?
    - How did HIV status and antiretroviral treatment interact with the time-restricted eating intervention?

4. Data Collection Challenges

    - What were the most difficult aspects of collecting accurate data over 12 months?
    - How did you manage participant retention throughout the study?
    - Can you describe any unexpected obstacles in data collection?

5. Participant Support and Communication

    - What communication strategies were most effective in maintaining participant motivation?
    - How did you address participant concerns or misconceptions about the intervention?
    - What type of support did participants seem to need most during the study?

6. Observations and Insights

   - What surprised you most about participants' experiences with time-restricted eating?
   - Did you observe any unexpected health or lifestyle changes among participants?
   - What recommendations would you make for similar future studies?

7. Personal Reflection

   - How has this experience impacted your understanding of nutrition interventions?
   - What did you learn about conducting research in this community?
